# Neuropsychological effects of COVID‐19: A review

**DOI:** 10.1002/brb3.2602

**Published:** 2022-07-28

**Authors:** Giuseppa Maresca, Desiree Latella, Lara Carnazza, Francesco Corallo, Caterina Formica

**Affiliations:** ^1^ IRCCS Centro Neurolesi Bonino‐Pulejo Messina Italy

**Keywords:** COVID‐19, emergency psychology, emotional epidemiology, mental health, psychological intervention

## Abstract

**Objective:**

The purpose of this review is to examine review literature on the psychological effects of the COVID‐19 pandemic.

**Methods:**

Studies were identified by an online search of the PubMed database. We selected studies published from January to May 2020 (during the COVID‐19 emergency).

**Results:**

We found that psychological effects of COVID‐19 remain serious among the most of the population, in particular for people with mental disorders, adolescents, healthcare workers, and the general population that experienced high levels of stress, anxiety, and depression symptoms, with possible long‐term psychological implications.

**Conclusion:**

Findings revealed that living in urban areas, having economic stability, and living with parents were protective factors against anxiety for youth groups, whereas a risk factor was represented by the presence of COVID‐19 infection that involved family members.

## Graphical abstract

The purpose of this review is to examine the psychological effects of the COVID‐19 pandemic. For this reason, we selected articles from January to March 2020 from PubMed database. We focused on the psychological effects of specific population: people with mental disorders, adolescents, healthcare workers, and the general population that experienced high levels of stress, anxiety, and depression symptoms. Findings revealed that living in urban areas, having economic stability, and living with parents were protective factors against anxiety for youth groups, whereas a risk factor was represented by the presence of COVID‐19 infection that involved family members.

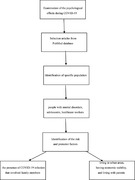

## 1 INTRODUCTION

In March 2020, the coronavirus pandemic (COVID‐19) paralyzed the whole world causing many victims. It represents an event with a strong impact in the life of each individual, at many levels (personal, work, social, economic, and psychological). The emergency puts a strain on our psychological health, fueling concerns and uncertainty, due to the daily update of data on the infection and lethality of the virus and on the expansion of the virus (Torales et al., 2020). However, awareness of the devastating impact that irresponsible communication can have on the psychological aspects of the community has slowly increased in the general public. Fear of disease and uncertainty about the future trigger anxiety disorders and distress; the creation and dissemination of screening and treatment programs for the mental health of patients and health professionals becomes necessary (Ali & Alharbi, 2020). COVID‐19 pandemic psychologically affected the total general population. In particular, the psychological impact of the epidemic on the general public, patients, medical staff, children, and older adults was significant (80%) (Li, Guan, et al., 2020). To date, few studies about the mental health status of students facing the epidemic have been conducted. The results indicated that 24.9% of college students experienced anxiety because of the COVID‐19 outbreak. Of these students, 0.9% experienced severe anxiety and 21.3% experienced mild anxiety (Tull et al., 2020). For specific population groups, the consequences were more devastating. One aspect to consider was the effect on people with mental health disorders. In fact, people with a weak health condition were more susceptible to stress than the general population characterized by an increase in fear, anxious, and depressive symptoms. In this perspective, a program of psychological interventions for the reduction of these symptoms becomes necessary (Yao et al., 2020). In this difficult situation of global emergency, important difficulties in management were represented by hospital and healthcare workers. Röhr et al. (2020) show that the psychological stress of health workers is often due to a divergence of roles; on the one hand, there is a sense of professional responsibility, and on the other, anxiety, worries, guilt, and fear of infecting family members. A study assessed the psychological stress and trauma caused by the COVID‐19 pandemic on 214 patients and 526 nurses, showing that more attention is needed to the psychological problems of healthcare professionals as well as prevention and treatment strategies for health care and for patients (Li et al., 2020). The purpose of this brief review is to investigate literature researches about the psychological effects of the COVID‐19 pandemic.

## 2 MATERIALS AND METHODS

### 2.1 Search strategy

Studies were identified by an online search of the PubMed database. We selected studies published from January to May 2020 (during the COVID‐19 emergency). The search combined the following terms: (“COVID‐19”[All Fields] OR “COVID‐2019”[All Fields] OR “severe acute respiratory syndrome coronavirus 2”[Supplementary Concept] OR “severe acute respiratory syndrome coronavirus 2”[All Fields] OR “2019‐nCoV”[All Fields] OR “SARS‐CoV‐2”[All Fields] OR “2019nCoV”[All Fields] OR “Wuhan”[All Fields] AND “coronavirus”[MeSH Terms] OR “coronavirus”[All Fields] AND 2019/12[PDAT] OR 2020[PDAT] AND psychological[All Fields] AND effects[All Fields]). Only English language articles were selected. There were a total of 1335 articles. The search terms were identified as title and abstract. All articles were evaluated by title, abstract, and text, after they fulfilled the following criteria: (1) research published with peer review; (2) studies including psychological effects caused by COVID‐19 pandemic; and (3) articles published before May 2020. We excluded articles in other languages and articles that discussed different fields of COVID‐19 such as articles about the SARS epidemic 2003.

## 3 RESULTS

**FIGURE 1:**
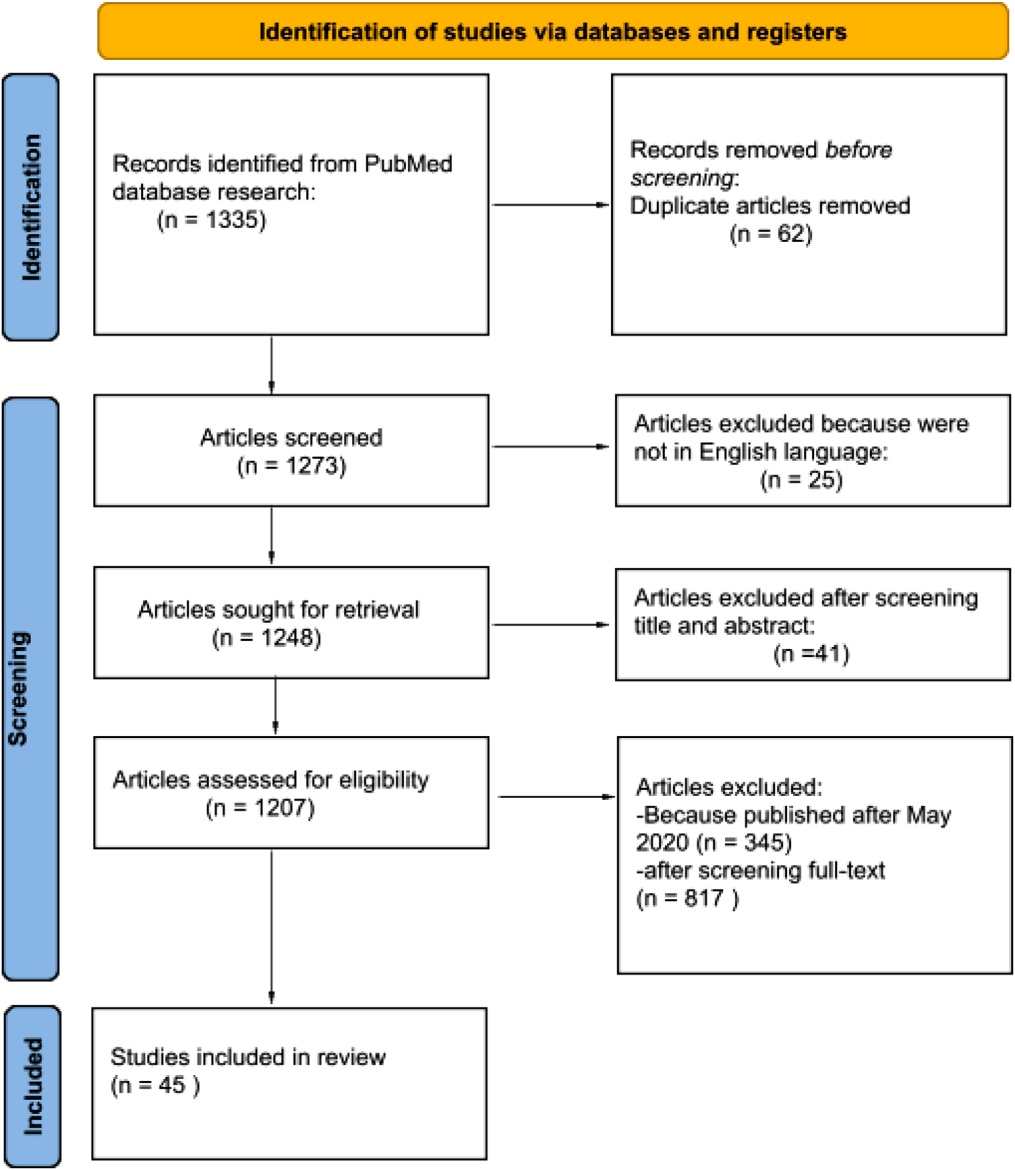
PRISMA 2020 flow diagram of evaluated studies

In total, 1335 articles were searched. Sixty‐two articles were removed after screening due to duplication. Forty‐one articles were excluded based on the screening of titles and abstracts. Three hundred and forty‐five articles were removed because they were published after May 2020. Twenty‐five articles were excluded because they were not in English language. Eight hundred and seventeen articles were excluded after screening full text. Forty‐five research articles met the inclusion criteria (Figure 1). Articles described in this review investigated the psychological aspects in various groups of people and we also considered populations of different nationalities especially including China, Pakistan, Germany, Spain, Italy, the United States, and Turkey. In our review, we considered the papers in the acute period of COVID‐19 pandemic. In particular, we selected articles about the psychological consequences of healthcare workers caused by the massive hospitalizations (Cai et al., 2020; Cole et al., 2020; Hou et al., 2020; Kisely et al., 2020; Wu et al., 2020; Xiao et al., 2020; Yin et al., 2020). Symptoms of post traumatic stress disorder (PTSD) were evaluated in two articles (Dutheil et al., 2021; Yin et al., 2020), and psychological impact in adolescents was also evaluated in two other articles (Buzzi et al., 2020; Liu et al., 2020). The psychological distress in adult patients was evaluated in five studies. In adults (Guo et al., 2020; Heitzman, 2020; Li, Wang, et al., 2020; Liu et al., 2020; Yang & Ma, 2020), psychological impact in people with a specific clinical conditions (DeJong et al., 2020), about social isolation and quarantine (Brooks et al., 2020; Razai et al., 2020; Satici et al., 2020), five articles discussed about global mental health in the general population. We also reported articles about the modality of intervention to promote psychological well‐being (Inchausti et al., 2020; Renjun et al., 2020; Van Bavel et al., 2020) such as the use of “teleconsulting” (Goodman‐Casanova et al., 2020).

Symptoms of depression and anxiety were described in most of the studies that we discussed (Gonzalez‐Sanguino et al., 2020; Mazza et al., 2020; Reger et al., 2020; Wang et al., 2020), including the general population, healthcare workers, and the weak population. Studies revealed that living in urban areas, having economic stability, and living with parents represented protective factors against anxiety and depressive symptoms, whereas a risk factor was represented by the presence of COVID‐19 infection that involved family members (Gonzalez‐Sanguino et al., 2020; Mazza et al., 2020). Results showed that depression, anxiety, stress, and posttraumatic stress disorder are significantly associated with the presence of physical symptoms (Chew et al., 2020). In particular, the incidence of anxiety was 26.60% (mild, 23.19%; moderate, 2.71%; severe, 0.70%), whereas depressive symptoms were detected in 21.16% of the students (mild, 16.98%; moderate, 3.17%; moderate‐to‐severe, 1.01%) (Buzzi et al., 2020; Liu et al., 2020). No significant differences were revealed between male and female students in terms of distress and negative emotions experienced (Moreno et al., 2019). About the healthcare workers, Nelson et al. (2020) showed the prevalence of loneliness in 38.5%, anger in 28.6%, and fear in 22.4%. Liu and colleagues (2020) reported anger in 16.6% and anxiety in 7.6% of quarantined respondents in hospital staff. A recent study conducted in China by Wang et al. (2020) showed that most of the 1210 respondents spent 20–24 h a day at home (84.7%), worried about their family members (75.2%), and were satisfied with the amount of health information available (75.1%). A study assessed the psychological stress and trauma caused by the COVID‐19 pandemic on 214 patients and 526 nurses, showing that more attention is needed to the psychological problems of healthcare professionals as well as prevention and treatment strategies for health care and for patients (Li et al., 2020).

## 4 DISCUSSIONS

Our review explored the effects of COVID‐19 pandemic on the psychological status in the general population and specific range of people such as healthcare professionals, people with weak health conditions, and young people. We observed that there is a higher prevalence of psychological symptoms and their variations in prevalence depend on different populations. In fact, in this critical situation, healthcare professionals directly involved in the diagnosis, treatment, and care of patients with COVID‐19 are at risk of developing psychological distress and other mental disorders (Lai et al., 2020; Sjodin et al., 2020).

An increase in confirmed and suspected cases, workload, exhaustion of personal protection equipment, outbreaks, lack of specific drug therapy, and adequate support can all contribute to increasing the stress of workers.

In fact, Lai et al. (2020) show that 1257 Wuhan health workers, engaged in the front line in the management of the first patients with COVID‐19, suffered psychological overload with symptoms of depression, anxiety, insomnia, and distress. Furthermore, Röhr et al. (2020) show that the psychological stress of health workers is often due to a divergence of roles; on the one hand, there is a sense of professional responsibility, and on the other, anxiety, worries, guilt, and fear of infecting family members.

The continuous spread of the epidemic, strict isolation measures and delays in starting schools, colleges, and universities internationally influenced the mental health of students. Institutions have therefore equipped themselves with online learning platforms. Many faculty members trained themselves to use online learning platforms either as the only delivery mode or as an add‐on to face‐to‐face teaching (Zhai & Du, 2020). The psychological impact of the epidemic on the general public, patients, medical staff, children, and older adults was significant (80%) (Li et al., 2020). However, few studies about the mental health status of students facing the epidemic have been conducted to date. Methods of supporting students to effectively and appropriately regulate their emotions during public health emergencies and avoid losses caused by crisis events have become an urgent problem for educational institutions. In a survey conducted on 7143 medical students, many students reported anxiety, fear, and worry, among others (Cao et al., 2020).

Technology resources became more useful to provide psychological intervention during COVID‐19 pandemic. For this reason, telehealth may be effective and became part of our routine healthcare system. The spread of telehealth also requires a significant change in the management effort and the redesign of existing care models. Substantial efforts have been made to scale down the routine use of telemedicine, often with little success (Alwashmi, 2020). In Australia, despite the introduction of generous financial incentives for specialized video consultations, telehealth accounted for less than 1% of all specialist consultations provided (Hersh et al., 2001).

## 5 CONCLUSION

This review demonstrated that psychological effects of COVID‐19 remain serious especially in a weak population, such as people with mental disorders, youth groups, and healthcare workers. In particular, we identified protective factors such as living in urban areas, economic stability, and living with other relatives. During the COVID‐19 epidemic period, the clinicians should focus on the psychological impact of the epidemic on healthcare workers, weak populations, and young people. Future research should explore the other factors affecting mental health in public emergencies such as the COVID‐19, focus on providing strategies for stress tolerance, and promote resilience and psychological wellness.

## CONFLICT OF INTEREST

The authors declare no conflict of interest.

### PEER REVIEW

The peer review history for this article is available at https://publons.com/publon/10.1002/brb3.2602.

## Data Availability

Data sharing is not applicable to this article as no new data were created or analyzed in this study.
